# Correlation between serum liver fibrosis markers and early gastroesophageal varices among patients with compensated liver cirrhosis: a cross-sectional analysis

**DOI:** 10.1186/s12876-022-02546-w

**Published:** 2022-12-12

**Authors:** Ling Mei, Ying Ma, Lili Zhao, Qingling Chen, Li Zhou, Hang Yang, Jie Liu, Jia Li

**Affiliations:** 1 Department of Gastroenterology and Hepatology, Clinical School of the Second People’s Hospital, Tianjin Medical University, Tianjin, China; 2 Department of Hepatology, Tianjin Second People’s Hospital, No. 7, Sudi South Road, Nankai District, Tianjin, 300192 China

**Keywords:** Early gastroesophageal varices, Endoscopic ultrasonography, Hyaluronic acid, Laminin, Type III procollagen, And type IV collagen

## Abstract

**Background and aim:**

Portal hypertension is a common complication of chronic liver diseases responsible for most liver cirrhosis consequences. In patients with portal hypertension, oesophagogastric variceal bleeding is a leading cause of death. Most research has focused on high-risk gastroesophageal varices and bleeding, with only a few studies on early varices. However, early intervention of gastroesophageal varices was found to better improve the prognosis and reduce mortality, but there is still no relevant research. Ultrasonic endoscopy is a combination of endoscopy and ultrasonic imaging. It can gastroscopically detect varices around the oesophagus and stomach and detect oesophageal collateral veins and perforating veins earlier, which is helpful for the early diagnosis of varices. Therefore, this study aimed to explore the correlation between serum fibrosis markers and early gastroesophageal varices in compensated cirrhosis patients.

**Methods:**

This study included 791 patients with compensated cirrhosis. The selected patients were categorized into three groups. The early gastroesophageal varices group included patients with gastroesophageal varices found by endoscopic ultrasonography but not by gastroscopy. The no gastroesophageal varices group underwent endoscopic ultrasonography and gastroscopy without varices. In addition, gastroesophageal varices can be detected with both techniques. Multiple logistic regression analysis explored the association of serum fibrosis markers with early gastroesophageal varices.

**Results:**

Among the 791 compensated liver cirrhosis patients, 198 patients were without gastroesophageal varices, 279 patients had early gastroesophageal varices, 314 patients had gastroesophageal varices, and both techniques could detect varices. There was a positive correlation between serum fibrosis markers and early gastroesophageal varices. In univariate logistic regression analysis, the patients with early gastroesophageal varices had lower platelet counts (*P* = 0.034) and higher aspartate aminotransferase (*P* = 0.046), total bilirubin (*P* = 0.041), hyaluronic acid (*P* < 0.001), laminin (*P* < 0.001), type III procollagen (*P* = 0.005), type IV collagen (*P* = 0.002), liver stiffness measurement (*P* = 0.001), APRI (*P* = 0.019) and FIB-4 (*P* = 0.002). Multivariate analysis showed that laminin (OR 1.011; 95% CI 1.004-1.017, *P* = 0.001) was an independent risk factor for predicting early gastroesophageal varices in compensated cirrhosis patients.

**Conclusion:**

Higher laminin was independently associated with early gastroesophageal varices in compensated cirrhosis patients.

## Introduction

Portal hypertension, defined as increased pressure in the portal venous system, is a major complication of liver cirrhosis [1]. Vasodilation of the splanchnic capillary beds and arterioles increases the portal blood flow, which, combined with an increase in the intrahepatic vascular resistance, increases the portal pressure [2]. Its complications, which include ascites, gastroesophageal varices, hepatic encephalopathy, and hepatorenal syndrome, cause significant morbidity and mortality [3]. Collaterals develop at the sites of communication between the portal and systemic circulation when the portal pressure increases above a threshold. The most significant collaterals are gastroesophageal varices [4, 5]. Anatomic studies have revealed that early gastroesophageal varices (GEVs) are dilated deep intrinsic veins in the submucosa of the distal oesophagus and proximal stomach of patients with portal hypertension [6, 7]. Patients with varices have a one-third probability of developing a variceal bleed 2 years after diagnosis, with a 20 to 40% mortality rate per episode [8]. Gastroesophageal variceal bleeding is a leading cause of death in patients with cirrhosis [9]. Therefore, early detection of early gastroesophageal varices significantly reduces mortality and medical costs and improves patient survival and prognosis. However, most current studies have focused on high-risk oesophageal and gastric varices and bleeding-related research.

Cirrhotic patients must undergo routine gastroscopy. However, the scope of gastroscopy is limited to the intramucosal and submucosal blood vessels, and the haemodynamic changes in cirrhotic patients with portal hypertension is not understood. Endoscopic ultrasound (EUS) is a noninvasive method that can provide high-resolution anatomic images and can be used to determine the haemodynamic features of collateral vessels surrounding the distal oesophagus and upper stomach in patients with portal hypertension [10]. Therefore, when judging the changes in the collateral circulation of patients with portal hypertension, we can observe the oesophageal and gastric varices that can be seen under conventional gastroscopy and detect the vascular lesions inside and outside the oesophageal wall that cannot be seen under standard endoscopy. EUS can objectively be used to visualize gastroesophageal varices (GEVs) and is more sensitive than conventional endoscopy in detecting GEVs [6, 11, 12]. In Gin-Ho Lo’s study, EUS detected gastric varices (GV) in 28 patients (35%), while in routine endoscopy, only 6 patients (7%) were found to have GV who were noted to have GV by conventional endoscopy [13]. Changjun men et al. reported that a patient with hepatitis B cirrhosis, whose varices were not detected by plain endoscopy and whose oesophageal and gastric varices were detected by EUS, developed oesophageal and gastric variceal bleeding 6 months after the examination [14]. EUS is widely accepted as helpful in assessing oesophagogastric varices because it provides good delineation of the cross-sectional anatomy of the distal oesophagus and proximal stomach and can detect gastroesophageal varices along with periesophageal collateral veins, paraesophageal collateral veins, and perforating veins; therefore, EUS can detect gastroesophageal varices earlier than gastroscopy [15, 16]. Previous studies have also shown that EUS has more advantages in detecting oesophageal and gastric varices caused by portal hypertension and early liver fibrosis [6, 17]. According to Y. T. Lee’s research, the advantage of EUS is that it can provide information about venous abnormalities at the gastroesophageal junction due to the elevated portal vein pressure in patients with early cirrhosis [6]. In recent years, EUS technology has developed rapidly, and EUS has become more critical in diagnosing and treating portal hypertension-related varices. However, this examination is expensive, time-consuming, difficult to perform and has high technical requirements, and the patient can also feel pain during the test. It is difficult to repeat the examination and pursue long-term follow-up, and it cannot be widely popularized in clinical practice. The Baveno VII portal hypertension consensus suggests that the possibility of high-risk varices can be confirmed by liver stiffness and platelet tests to avoid unnecessary endoscopy [18]. However, no alternative noninvasive markers have been found for early portal hypertension. Therefore, it is of great significance to find a noninvasive marker to evaluate oesophageal and gastric varices in patients with compensatory cirrhosis.

Serum liver fibrosis markers include hyaluronic acid (HA), laminin (LN), type III procollagen, and type IV collagen. These four markers of liver fibrosis have been proven to be highly correlated with liver fibrosis in patients with chronic liver disease [19, 20]. It has been demonstrated that liver fibrosis markers have high predictive value for the diagnosis and prognosis of patients with chronic liver disease [21–23]. However, there are still few studies on the relationship between serum liver fibrosis markers and the severity and complications of oesophageal and gastric varices. Therefore, we conducted a retrospective cross-sectional study to evaluate the correlation between indicators of liver fibrosis and early gastroesophageal varices in patients with compensatory cirrhosis.

## Methods

### Study population

In this retrospective study, 791 patients with compensated cirrhosis were enrolled between November 2015 and December 2019. Eligible patients aged 18 years or older who were diagnosed with compensated cirrhosis according to imaging studies using ultrasonography, computed tomography (CT), or magnetic resonance imaging (MRI) and who had an irregular and nodular liver together with impaired liver synthetic function could receive a diagnosis of cirrhosis [24]. All patients completed oesophagogastroduodenoscopy (EGD), EUS, FibroScan, imaging, and laboratory examinations within 6 months before and after hospitalization. The exclusion criteria were as follows: 1. primary prevention with nonselective β-blockers or endoscopic ligation; 2. thrombosis of the cavernous transformation of the portal vein system; 3. history of malignancy (including hepatocellular carcinoma); and 4. incomplete clinical data.

This study protocol was approved by the Ethics Committee of Tianjin Second People’s Hospital and conformed to the 1975 Declaration of Helsinki principles. Informed consent was obtained from all patients. All methods were performed following relevant guidelines and regulations.

### General characteristics and laboratory investigations

Demographic data (age and sex) and laboratory results (routine blood tests, biochemistry tests, and immunology tests) were collected and analysed. Blood samples were collected 1 week before EUS and EGD. Albumin (ALB), aspartate aminotransferase (AST), serum alanine aminotransferase (ALT), and total bilirubin (TBIL) were detected by a Hitachi 7180 Automatic Biochemical Analyser (Hitachi, Ltd., Tokyo, Japan). According to the manufacturer’s recommendation, platelets (PLTs) were measured using a Sysmex XN-2000 haematology analyser (Sysmex Corporation, Kobe, Japan). Prothrombin time (PT) was performed by the clotting method on the automatic coagulometer “STAGO Compact” (“Diagnostica Stago,” France).

Serum markers of liver fibrosis were measured with AutoLumo A2000 Plus. The upper reference value of laminin ln was 130 ng/ml, the upper reference value of type III procollagen was 15 ng/ml, the upper reference value of hyaluronic acid was 120 ng/ml, and the upper reference value of type IV collagen was 95 ng/ml. The coefficient of variation CV (%) did not exceed 15%.

### Assessment of varices

Two experienced operators performed the EGD using the equipment (Olympus CV-260SL, Japan). The variance was described according to the expert guidelines [24]. EUS was conducted by skilled professional doctors using the equipment (Olympus GF-UE260, Japan). According to the results of gastroscopy and endoscopic ultrasonography, the patients were divided into three groups: no gastroesophageal varices (negative on EUS and EGD); early gastroesophageal varices (positive on EUS and negative for EGD), and positive on EUS and EGD. In this study, we mainly focused on comparing the first two groups.

### Transient elastography

Liver stiffness (LS) was measured by transient elastography (Echosens, Fibroscan 520, Paris, France) by two experienced practitioners with professional training. The right lobe of the liver was accessed while the patient was positioned with maximal right arm abduction during the operation. The measurements were considered valid if ten measurements were obtained with a 60% success rate of all the total measurements and with an interquartile range of less than 30%.

### Spleen size

The patients fasted overnight and routinely underwent abdominal ultrasound examinations to measure the spleen size. Spleen diameters were reported and recorded. Ultrasound was performed by professional operators who did not know the clinical information about the patients.

### Statistical analysis

SPSS statistical 26 (IBM, New York, USA) and MedCalc Statistical Software version 15.8 (MedCalc Software bvba, Ostend, Belgium; https://www.medcalc.org; 2015). *P* < 0.05 was considered statistically significant. The Kolmogorov–Smirnov test was used to analyse whether continuous variables obeyed a normal distribution. The Kruskal–Wallis test was used for comparing nonparametric variables. The nonnormally distributed variables are expressed as the median (quartile 25, 75). The data for categorical variables are presented as the number (n) and proportion (%). The correlation between variables and early gastroesophageal varices was evaluated by Spearman correlation coefficient (r), chi-square test, and binary logistic regression analysis. The “enter” method was used for univariate logistic regression, and the statistically significant variables were included in the univariate logistic regression analysis. The receiver operating characteristic (ROC) curve of laminin was analysed for the prediction of early gastroesophageal varices by the DeLong method.

## Results

### General characteristics of the patients

A total of 791 compensated cirrhosis patients with a median age of 52 years were included in this study. In total, 716 (90.5%) patients were Child–Pugh class A, and 75 (9.5%) were Child–Pugh class B. According to the different aetiological classifications, there were 561 patients with hepatitis B (71%), 111 patients with hepatitis C (14%), and 119 patients with other liver diseases (15%). Further demographic data and laboratory blood markers of all patients are shown in Table 1.

**Table 1:** General characteristics of patients with compensated liver cirrhosis

Variables	*n =* 791
Age (years)	52 (42，59)
Male, n(%)	468 (59.2%)
Aetiology of cirrhosis, n(%)	
HBV	561 (71%)
HCV	111 (14%)
others	119 (15%)
Child category, n(%)	
A	716 (90.5%)
B	75 (9.5%)
ALT(U/L)	51 (26, 123)
AST(U/L)	46 (27, 103)
TBIL (umol/L)	17.4 (13.2, 24.4)
ALB(g/L)	42.6 (38.8, 45.9)
PLT(× 10^9^/L)	136 (99, 173.5)
PT(s)	13.6 (12.8, 14.6)
Hyaluronic acid (ng/ml)	148 (93, 332)
Laminin (ng/ml)	96 (86, 129)
type III pro-collagen (ng/ml)	9.8 (7, 14)
Collagen type IV (ng/ml)	78 (63, 154)
LS (kPa)	15.1 (9, 25.7)
Splenic diameter (mm)	114 (102, 126)
APRI	1 (0.5, 2.4)
FIB-4	2.73 (1.56, 5.05)

Data are expressed as median (quartile 25, quartile 75) or number (proportion)

ALT alanine aminotransferase, AST aspartate aminotransferase, TBIL total bilirubin, ALB albumin, PLT platelet, PT prothrombin time, LS liver stiffness, APRI: Aspartate aminotransferase-to-Platelet Ratio Index, FIB-4: Fibrosis 4 Score

### General characteristics of the patients by gastroesophageal variceal severity

The included patients were divided into the non-GEV group by EUS(−) EGD(−), the early GEV group by EUS(+) EGD(−), and the EUS(+) EGD(+) group. Among the 791 patients, 314 (39.7%) patients with EUS(+) EGD(+), 279 (35.3%) had early GEVs, and 198 (25%) did not. We found that with the development of gastroesophageal varices, the total bilirubin (TBIL), liver stiffness (LS), serum liver fibrosis markers (hyaluronic acid, laminin, type III procollagen, and type IV collagen), aspartate aminotransferase-to-platelet ratio index (APRI) and fibrosis 4 score (FIB-4) were also increased significantly, as shown in Table 2. In this study, we mainly focused on the first two groups. TBIL, LS, APRI, FIB-4, and serum liver fibrosis markers were significantly higher in the early GEV group than those in the non-GEV group (*P* < 0.05), while PLT was significantly lower in the early GEV groups than that in the non-GEV group (*P* < 0.05). There was no significant difference in the other indexes between the first two groups (*P* > 0.05), as shown in Table 2.

**Table 2:** Characteristics of gastroesophageal varices of different severities

Variables	EUS-EGD-(*n =* 198)	EUS + EGD-(*n =* 279)	EUS + EGD+(*n =* 314)	P
Age (years)	50 (39.75, 56.5)	51 (40, 57)	54 (44, 60)	<0.001
Male, n(%)	105 (53)	158 (60)	205 (65)	0.013
Aetiology of cirrhosis (HBV/HCV/other)	145/26/27	217/34/28	199/51/64	0.002
Child category (A/B)	189/9	253/26	274/40	0.009*
ALT(U/L)	46 (24, 116)	57 (28, 164)	47 (26, 116)	0.231
AST(U/L)	42.5 (24, 86)	45 (28, 120)	53 (28, 108)	0.095
TBIL (umol/L)	15.2 (11.6, 20.725)	16.1 (13, 23.8)	18.8 (14.4, 27.8)	<0.001*
ALB(g/L)	43.8 (39.5, 46.425)	42.7 (39.3, 46.4)	41.4 (37.8, 45.2)	<0.001
PLT(×10^9^/L)	152 (120.5, 180.5)	137 (105, 176)	115 (81, 161)	<0.001**
PT(s)	13.4 (12.7, 14.0)	13.5 (12.8, 14.4)	13.9 (12.8, 15.1)	<0.001*
Hyaluronic acid (ng/ml)	106.5 (87, 192.25)	141 (93, 322)	201 (98, 430)	<0.001**
Laminin (ng/ml)	91 (68.75, 98.0)	97 (89, 130)	102 (91.8, 146)	<0.001***
type III pro-collagen (ng/ml)	6.5 (9.0, 12.0)	9.7 (7.2, 16)	10 (7, 16)	0.001*
Collagen type IV (ng/ml)	55 (68, 114.75)	72 (62, 137)	102 (65, 183.3)	<0.001**
LS (kPa)	11.55 (7.675, 17.35)	14.2 (8.8, 23.2)	19.4 (10.8, 29.1)	<0.001**
Splenic diameter (mm)	110.5 (99.25, 120.75)	114 (99, 125)	118 (105, 133)	<0.001
APRI	0.79 (0.39, 1.49)	0.86 (0.5, 2.5)	1.27 (0.61, 2.91)	<0.001*
FIB-4	2.07 (1.23, 3.53)	2.36 (1.56, 4.51)	3.62 (2.03, 7.03)	<0.001**

Data are expressed as median (quartile 25, quartile 75) or number (proportion)

ALT alanine aminotransferase, AST aspartate aminotransferase, TBIL total bilirubin, ALB albumin, PLT platelet, PT prothrombin time, LS liver stiffness, APRI: Aspartate aminotransferase-to-Platelet Ratio Index, FIB-4: Fibrosis 4 Score

* *P<* 0.05, ** *P<* 0.01, *** *P<*0.001, between EUS-EGD- group and EUS + EGD- group

### Correlations between serum liver fibrosis markers and the clinical characteristics of patients with negative EGD

The correlations between the serum liver fibrosis marker variables and clinical characteristics are summarized in Table 3. As expected, the serum liver fibrosis markers were positively correlated with ALT, AST, TBIL, PT, LS, APRI, FIB-4, and Child–Pugh (C-P) classification (*P* < 0.05) and negatively correlated with ALB and PLT (*P* < 0.05). The length and diameter of the spleen were positively correlated with hyaluronic acid, type III procollagen, and type IV collagen (*P* < 0.05). Hyaluronic acid, laminin, type III procollagen, and type IV collagen were positively correlated with early gastroesophageal varices (*P* < 0.05).

**Table 3:** Correlations between serum fibrosis markers and clinical characteristics of patients with negative EGD

Variables statistics	Hyaluronic acid (ng/ml)		Laminin (ng/ml)		Type III pro-collagen (ng/ml)		Collagen type IV (ng/ml)	
	r	P	r	P	r	P	r	P
Age (years)	0.168	<0.001	0.006	0.895	−0.131	0.004	−0.116	0.011
Child class (A/B)	0.273	<0.001	0.216	<0.001	0.301	<0.001	0.291	<0.001
ALT(U/L)	0.210	<0.001	0.254	<0.001	0.439	<0.001	0.420	<0.001
AST(U/L)	0.331	<0.001	0.352	<0.001	0.518	<0.001	0.529	<0.001
TBIL (μmol/L)	0.266	<0.001	0.290	<0.001	0.289	<0.001	0.256	<0.001
ALB(g/L)	−0.401	<0.001	−0.302	<0.001	−0.431	<0.001	−0.494	<0.001
PLT(×10^9^/L)	−0.314	<0.001	−0.249	<0.001	−0.150	<0.001	−0.267	<0.001
PT(s)	0.212	<0.001	0.217	<0.001	0.300	<0.001	0.339	<0.001
LS (kPa)	0.484	<0.001	0.430	<0.001	0.469	<0.001	0.568	<0.001
Splenic diameter (mm)	0.149	0.001	0.088	0.059	0.159	0.001	0.204	<0.001
APRI	0.404	<0.001	0.397	<0.001	0.357	<0.001	0.565	<0.001
FIB-4	0.496	<0.001	0.387	<0.001	0.521	<0.001	0.445	<0.001
EGD(−)EUS(−/+)	0.142	0.002	0.253	<0.001	0.130	0.004	0.127	0.006

*ALT* Alanine aminotransferase, *AST* Aspartate aminotransferase, *TBIL* Total bilirubin, *ALB* Albumin, *PLT* Platelet, *PT* Prothrombin time, *LS* Liver stiffness, *APRI* Aspartate aminotransferase-to-Platelet Ratio Index, FIB-4: Fibrosis 4 Score

EGD (−)Esophagogastroduodenoscopy negative， *EUS*(−/+) Endoscopic ultrasound negative or positive

### Analysis of risk factors associated with early GEVs

Binary logistic regression analysis explored the risk factors associated with early GEVs in patients with compensated cirrhosis. In the univariate analysis, the patients with early portal hypertension had lower PLT (*P* = 0.034), higher AST (*P* = 0.046), TBIL (*P* = 0.041), HA (*P* < 0.001), LN (*P* < 0.001), procollagen III (*P* = 0.005), collagen IV (*P* = 0.002), LS (*P* = 0.001), APRI (*P* = 0.019) and FIB-4 (*P* = 0.002). However, after the multivariate logistic regression analysis, it was found that LN was an independent risk factor for early GEVs in patients with compensated cirrhosis, with an odds ratio (OR) (95% confidence interval CI) of 1.011 (1.004, 1.018), *P* = 0.001. as shown in Table 4.

**Table 4:** Univariate and multivariate analysis of factors associated with early gastroesophageal varices

Variables	Univariate analysis		Multivariate analysis	
	OR(95%CI)	P	OR(95%CI)	
Age (years)	1.004 (0.989, 1.020)	0.612		
Male, n(%)	0.865 (0.600, 1.247)	0.436		
Aetiology of cirrhosis (HBV/HCV/other)	0.839 (0.643, 1.095)	0.197		
Child category (A/B)	2.158 (0.988, 4.713)	0.054		
ALT(U/L)	1.001 (1.000, 1.001)	0.246		
AST(U/L)	1.001 (1.000, 1.002)	0.046		
TBIL (umol/L)	1.008 (1.000, 1.016)	0.041	1.003 (0.995, 1.011)	0.492
ALB(g/L)	0.981 (0.949, 1.015)	0.247		
PLT(×10^9^/L)	0.996 (0.993, 1.000)	0.034		
PT(s)	1.056 (0.971, 1.149)	0.205		
Hyaluronic acid (ng/ml)	1.001 (1.001, 1.002)	<0.001	1.000 (0.999, 1.001)	0.567
Laminin (ng/ml)	1.015 (1.009, 1.020)	<0.001	1.011 (1.004, 1.018)	0.001
type III pro-collagen (ng/ml)	1.040 (1.012, 1.069)	0.005	1.001 (0.966, 1.037)	0.964
Collagen type IV (ng/ml)	1.004 (1.001, 1.006)	0.002	0.999 (0.995, 1.002)	0.436
LS (Kpa)	1.032 (1.113, 1.051)	0.001	1.012 (0.987, 1.037)	0.348
Splenic diameter (mm)	1.003 (0.993, 1.013)	0.569		
APRI	1.080 (1.012, 1.151)	0.019	0.976 (0.886, 1.075)	0.618
FIB-4	1.121 (1.042, 1.206)	0.002	1.036 (0.924, 1.161)	0.544

*ALT* Alanine aminotransferase, *AST* Aspartate aminotransferase, *TBIL* Total bilirubin, *ALB* Albumin, *PLT* Platelet, *PT* Prothrombin time, *LS* Liver stiffness, *APRI* Aspartate aminotransferase-to-Platelet Ratio Index, *FIB-4* Fibrosis 4 Score

### Analysis of the diagnostic performance of laminin using the ROC curve

**Fig. 1:**
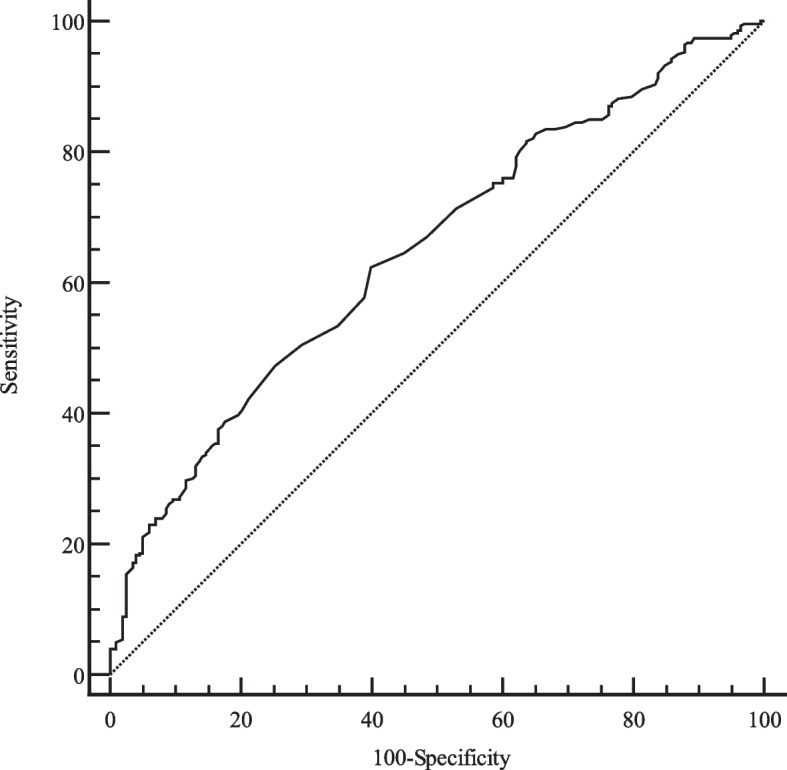
Receiver operating characteristic curve for Laminin patients with early gastroesophageal varices

The present study showed that laminin was an independent risk factor for early GEVs in patients with compensated cirrhosis. Then, to evaluate the diagnostic efficacy of laminin, we conducted a ROC curve analysis. The ROC curve (Fig. 1) showed that the best cut-off value for laminin was 93 ng/ml, with a 62.37% sensitivity, a 60.10% specificity, a positive predictive value (PPV) of 68.78%, and a negative predictive value (NPV) of 53.12% for the diagnosis of early GEVs (area under the curve [AUC] = 0.648, 95% CI 0.604-0.691; Table 5). Hence, we demonstrate that laminin levels were significantly associated with early gastroesophageal varices in patients with compensated cirrhosis, but the laminin levels might be inappropriate for diagnosing early GEVs.

**Table 5:** Performance of laminin for the prediction of early gastroesophageal varices

Variables	AUC(95%CI)	Cut-off value	Sensitivity (%)	Specificity (%)	PPV (%)	NPV (%)
Laminin (ng/ml)	0.648 (0.604-0.691)	93	62.37	60.10	68.78	53.12

*NPV* Negative predictive value, *PPV* Positive predictive value, *AUC* Area under the curve

## Discussion

Previous research has focused on identifying noninvasive serological indications of high-risk oesophagogastric varices to reduce unnecessary endoscopy in patients. Many studies have now shown that noninvasive indicators, including platelet count, albumin, spleen size, and liver stiffness, are related to the severity of high-risk oesophagogastric varices and cirrhosis [25–28]. Noninvasive indications of early oesophagogastric varices are the subject of comparatively few investigations. Therefore, this study aims to develop noninvasive indicators of oesophagogastric varices, decrease unnecessary gastroscopy and endoscopic ultrasonography, improve prognosis, and reduce mortality and medical expenditures.

This study conducted a correlation analysis between serum liver fibrosis markers and clinical indicators. First, we discovered that serum liver fibrosis markers are related to other indicators of gastroesophageal varices, liver stiffness, APRI, and Fib-4, suggesting that serum liver fibrosis markers could be used as a substitute for other indicators in the prediction of gastroesophageal varices. Second, we found a correlation between serum liver fibrosis markers and early gastroesophageal varices, demonstrating that an increase in serum liver fibrosis markers could indicate the presence of gastroesophageal varices. We also discovered a correlation between serum markers of liver fibrosis and C-P class, suggesting that serum markers of liver fibrosis are related to the severity of cirrhosis [29].

In this study, we found that hyaluronic acid, laminin, type III procollagen, and type IV collagen were positively correlated with early gastroesophageal varices in the univariate analysis and that the increase in laminin concentration was independently correlated with early gastroesophageal varices in compensated liver cirrhosis patients in the multivariate analysis after adjusting for confounding factors. Therefore, we discussed the diagnostic value of laminin and found that it has a limited diagnostic value for early gastroesophageal varices. We may need a prospective study to evaluate its diagnostic performance.

Chronic liver injury can result in fibrosis, characterized by the accumulation of extracellular matrix (ECM) in the liver. Hepatic stellate cells are activated by chronic liver cell injury. These cells acquire the fibrotic myofibroblast phenotype, resulting in collagen synthesis and per sinus contraction, leading to liver fibrosis and portal hypertension. Laminin is one of the major glycoproteins in the basement membrane. Hepatic stellate cells (HSCs) generate and deposit them in the liver’s basement membrane. The liver’s basement membrane is involved in biological tasks such as cell adhesion, matrix component interactions with collagen and glycosaminoglycans, cytoskeleton maintenance, and liver fibrosis [21, 30, 31]. The proportion of type I collagen, type III collagen, and laminin increases in the diseased state, while the mixture of laminin, type IV collagen, and proteoglycan increases in the ECM in normal liver [19, 30, 32]. According to several studies, serum liver fibrosis indicators are related to the severity of liver fibrosis caused by various aetiologies and are significant predictors of disease prognosis [33, 34]. In other research, the serum laminin concentration has also been related to gastroesophageal varices in cirrhosis. This study also found that the laminin concentration is related to early gastroesophageal varices in cirrhosis. Long Fei Wu’s study found that in a one-year prospective analysis, laminin and type IV collagen are possible predictors of rebleeding after oesophagogastric variceal surgery and are related to oesophageal and gastric varices [35].

This study has some limitations. First, this is a retrospective cross-sectional study, and we could not demonstrate a causal link between laminin and the risk of early oesophagogastric varices. A large-scale prospective survey is needed to further clarify the causal relationship between laminin and oesophagogastric varices. Not all patients received EUS examination and had their serum markers of liver fibrosis measured, which increases the possibility of bias. Second, liver biopsy is the gold standard for detecting liver fibrosis. However, invasive liver biopsy has some risks, including high technical requirements and low patient compliance. The patient may also experience pain during the examination. In addition, ultrasonography, CT, and MRI have been the most popular and well-validated noninvasive methods for assessing cirrhosis. Third, although there are standard procedures, there may be variability among the different observers.

In conclusion, we found that indicators of liver fibrosis were associated with early gastroesophageal varices, especially laminin, which was independently associated with early gastroesophageal varices in patients with compensatory cirrhosis. However, laminin has a limited diagnostic value for early gastroesophageal varices. Whether laminin can be used as a noninvasive reference index for monitoring gastroesophageal varices varies in patients with compensated needs to be further evaluated by a prospective study.

## Data Availability

The datasets used and/or analysed during the current study are available from the corresponding author upon reasonable request.

## Abbreviations

EUS: Endoscopic ultrasound
GEVs: Gastroesophageal varices
GV: Gastric varices
EGD: Esophagogastroduodenoscopy
HA: Hyaluronic acid
LN: Laminin
CT: Computed tomography
MRI: Magnetic Resonance Imaging
ECM: Extracellular matrix
HSCs: Hepatic stellate cells
ALT: Alanine aminotransferase
AST: Aspartate aminotransferase
TBIL: Total bilirubin
ALB: Albumin
PLT: Platelet
PT: Prothrombin time
LS: Liver stiffness
OR: Odds ratio
CI: Confidence interval
C-P: Child-pugh
APRI: Aspartate aminotransferase-to-Platelet Ratio Index
FIB-4: Fibrosis 4 Score
NPV: Negative predictive value
PPV: Positive predictive value
