# Correction to: Correlation between serum liver fibrosis markers and early gastroesophageal varices among patients with compensated liver cirrhosis: a cross-sectional analysis

**DOI:** 10.1186/s12876-023-02714-6

**Published:** 2023-03-30

**Authors:** Ling Mei, Ying Ma, Lili Zhao, Qingling Chen, Li Zhou, Hang Yang, Jie Liu, Jia Li

**Affiliations:** 1 Department of Gastroenterology and Hepatology, Clinical School of the Second People’s Hospital, Tianjin Medical University, Tianjin, China; 2 Department of Hepatology, Tianjin Second People’s Hospital, No. 7, Sudi South Road, Nankai District, Tianjin, 300192 China

**BMC Gastroenterology (2022) 22:515**

10.1186/s12876-022-02546-w

After publication of this article [1], the authors reported that in this article, Ying Ma should have been denoted as an equally contributing author instead of Jie Liu.

The original article [1] has been corrected.
